# Risk factors associated with blood dyscrasia during clozapine treatment: a systematic review and meta-analysis

**DOI:** 10.1017/S0033291726104371

**Published:** 2026-05-11

**Authors:** Cecilia Casetta, Emilia Loane, David Taylor, Richard Emsley, James Hunter MacCabe

**Affiliations:** 1 Institute of Psychiatry at the Maudsley: King’s College London Institute of Psyc, UK; 2 SLAM: South London and Maudsley NHS Foundation Trust, UK

**Keywords:** agranulocytosis, clozapine, blood dyscrasia, neutropenia, schizophrenia, treatment resistant psychosis

## Abstract

Clozapine is the only effective treatment for treatment-resistant schizophrenia, but concerns over blood dyscrasia and need for monitoring limit its use. Evidence suggests many hematological abnormalities may result from surveillance bias, with agranulocytosis being the primary adverse effect directly induced by clozapine. This systematic review (PROSPERO: CRD42024487199) investigates non-genetic risk factors associated with blood dyscrasia during clozapine treatment, focusing on neutropenia and agranulocytosis. Random-effect meta-analyses were performed on studies reporting quantitative risk data. Due to inconsistent neutropenia definitions, analyses used absolute neutrophil count (ANC) thresholds of <2000/mm^3^, <1500/mm^3^, and <500/mm^3^. Forty-four studies were included in the systematic review, 15 in meta-analyses. No significant association was found between agranulocytosis and female gender (OR = 1.48, 95% CI: 0.92–2.38; p = 0.106) or age (pooled standardized mean difference [SMD] = 0.32, 95% CI: –0.26 to 0.90, p = 0.285), whilst a modest inverse association with clozapine dose (SMD = –0.32, 95%CI: –0.50 to –0.14, p < 0.001) and baseline white cell count (SMD = –0.21, 95%CI: –0.40 to –0.03, p = 0.026) was found. Neutropenia (ANC < 2000/mm^3^) was positively associated with concomitant psychotropic use (OR = 2.15, 95% CI: 1.13–4.07, p = 0.019). Clozapine rechallenge studies revealed no significant associations with gender, age, duration of initial clozapine trial, or length of discontinuation period prior to rechallenge. No strong predictors of clozapine-associated blood dyscrasia were identified. Findings may be limited by study variability, surveillance bias, and lack of consistent differentiation between agranulocytosis and milder neutropenia, highlighting limitations in current evidence.

## Introduction

Clozapine is a unique antipsychotic and the only recommended treatment for treatment resistant schizophrenia (TRS) (National Institute for Health and Care Excellence, 2014). Despite its well documented efficacy (Stroup et al., 2016), its use is significantly limited by its side effect profile, especially the hematological reactions (Legge et al., 2016), which warrant strict blood count monitoring throughout treatment (Oloyede et al., 2022). This requirement often poses a barrier for both patients and prescribers, and it has been criticized as not being cost-effective (Girardin et al., 2014).

Clozapine has been associated with several types of blood dyscrasia, namely neutropenia, leukopenia, thrombocytopenia, eosinophilia and anemia. The most serious hematological side effect is severe neutropenia or agranulocytosis (absolute neutrophil count [ANC] <500/mm^3^), a rare but potentially fatal adverse event occurring in 0.4% of clozapine-treated patients, with a fatality rate of 0.05% (Li et al., 2020).

Extensive research has highlighted how the risk of agranulocytosis is higher in the first 18 weeks of treatment and becomes negligible after 2 years, with many experts arguing that the long-term monitoring requirements should be reviewed (Myles et al., 2018; Northwood et al., 2024; Rubio et al., 2024), a position recently endorsed in the updated EMA recommendations (European Medicine Agency, 2025). Conversely, mild episodes of neutropenia may occur at any point during clozapine treatment (Mena, Nachar, Crossley, & Gonzalez-Valderrama, 2019), and their causal association with clozapine remains unclear. These benign milder events are likely unrelated to agranulocytosis, which is considered an all-or-nothing phenomenon characterized by a rapid and profound reduction or near-complete absence of circulating neutrophils (Taylor et al., 2022).

The etiology of clozapine-induced agranulocytosis remains unclear; however, the prevailing hypothesis is that it is an immunological idiosyncratic reaction. Recent studies have investigated the role of the oxidation of clozapine to a reactive metabolite by myeloperoxidase, which seems critical for inducing the inflammatory response leading to severe neutropenia (Sernoskie, Jee, & Uetrecht, 2023; Wicinski & Weclewicz, 2018). In contrast, many mild neutropenic episodes and other blood dyscrasias observed during clozapine treatment may not be causally related to the drug and could reflect observation bias (Northwood et al., 2024). Supporting this, a within-subject study found no significant difference in neutropenia incidence during periods on and off clozapine, suggesting that mild cases may be entirely unrelated to clozapine exposure (Johannsen et al., 2022).

A recent meta-analysis reviewed genetic factors associated with clozapine-induced agranulocytosis and identified one genetic variant within the human leukocyte antigen (HLA) locus (Islam et al., 2022). While certain genetic variants appear to influence the risk of both neutropenia and agranulocytosis, recent findings have demonstrated a specific association between one specific HLA polymorphism and agranulocytosis, but not with neutropenia (Konte et al., 2021).

Identifying which individuals are at higher risk of developing agranulocytosis on clozapine would allow a more personalized monitoring approach, maximizing safety, whilst relaxing the monitoring requirements for those who are at low risk. Accordingly, research has focused on investigating potential risk factors and biomarkers of clozapine-associated blood dyscrasia, especially agranulocytosis.

This systematic review investigates non-genetic risk factors associated with blood dyscrasia during clozapine treatment, with a focus on neutropenia and agranulocytosis.

## Methods

This review was performed and reported within PRISMA guidelines (Page et al., 2021), following a pre-registered protocol (PROSPERO: CRD42024487199).

### Search strategy

Five electronic databases (Embase, Ovid, PsychINFO, Web of Science, and Scopus) were searched for research relating to risk factors for clozapine-associated blood dyscrasia. The initial database search was conducted on 13/01/2024. No filters were applied. Titles and abstracts returned from the search were screened for inclusion and exclusion criteria, and papers considered potentially eligible were full text screened by two independent reviewers (CC and EL), with disagreement resolved through discussion with a third reviewer (JM). Reference lists of articles meeting inclusion criteria were hand-searched, and all non-duplicate potential papers were full-text screened. To ensure the review was up to date, the search was rerun on 02/04/2025, including all studies from inception to 01/04/2025, and any additional eligible studies were screened and included using the same criteria.

### Inclusion and exclusion criteria

Studies published in peer-reviewed journals were included if they reported on demographic and clinical risk factors associated with any blood dyscrasia occurring during clozapine treatment. Study designs using prospective, retrospective and cross-sectional determination of clozapine-associated blood dyscrasia were all included if demographic and clinical measures were available from when the dyscrasia occurred. Reviews, case series, opinion papers, and anecdotal reports were excluded.

### Data extraction and quality assessment

Data extraction for included studies started on 14/05/2024. All data were independently extracted by two authors (CC and EL). Overall study quality was assessed using the Newcastle-Ottawa Scale. The assessments were independently performed by two reviewers (CC and EL), with disagreements resolved through discussion or the input of a third reviewer (Supplementary Table S1).

Data on study design and duration, study population, blood dyscrasia definition, and any demographic and clinical variables measured during clozapine treatment were extracted.

### Meta-analysis

Studies were eligible for meta-analysis if they reported quantitative information on risk factors for blood dyscrasia. Due to the variability in how neutropenia is defined across studies, it was not feasible to group studies based on the distinction between agranulocytosis and mild neutropenia. Consequently, we conducted multiple meta-analyses using the following ANC thresholds: ANC <2000/mm^3^, ANC <1500/mm^3^, and ANC <500/mm^3^. For studies that did not report odds ratios (ORs) but provided raw data and sample size, we calculated ORs manually using the available raw numbers. This allowed inclusion of additional data points in the meta-analysis and ensured a more comprehensive synthesis of the evidence.

Meta-analyses were performed using STATA 18 (StataCorp, 2023). A random-effects model was employed to account for potential heterogeneity across studies. Effect sizes were summarized using pooled ORs for binary outcomes and standardized mean differences (SMDs) for continuous outcomes. Pooled estimates were presented alongside their corresponding 95% confidence intervals (CIs). Forest plots were used to visually display the effect sizes of individual studies as well as the overall pooled effect. Heterogeneity was assessed using the *I* ^2^ statistic.

To assess the robustness of our meta-analytic findings, sensitivity analyses were conducted by repeating the analyses after excluding studies rated as low quality in the risk of bias assessment whenever sufficient studies remained after exclusion. This approach aimed to explore the extent to which study quality influenced the pooled estimates and to ensure that the overall conclusions were not unduly driven by methodologically weaker studies.

## Results

### Search results

**Figure 1.**
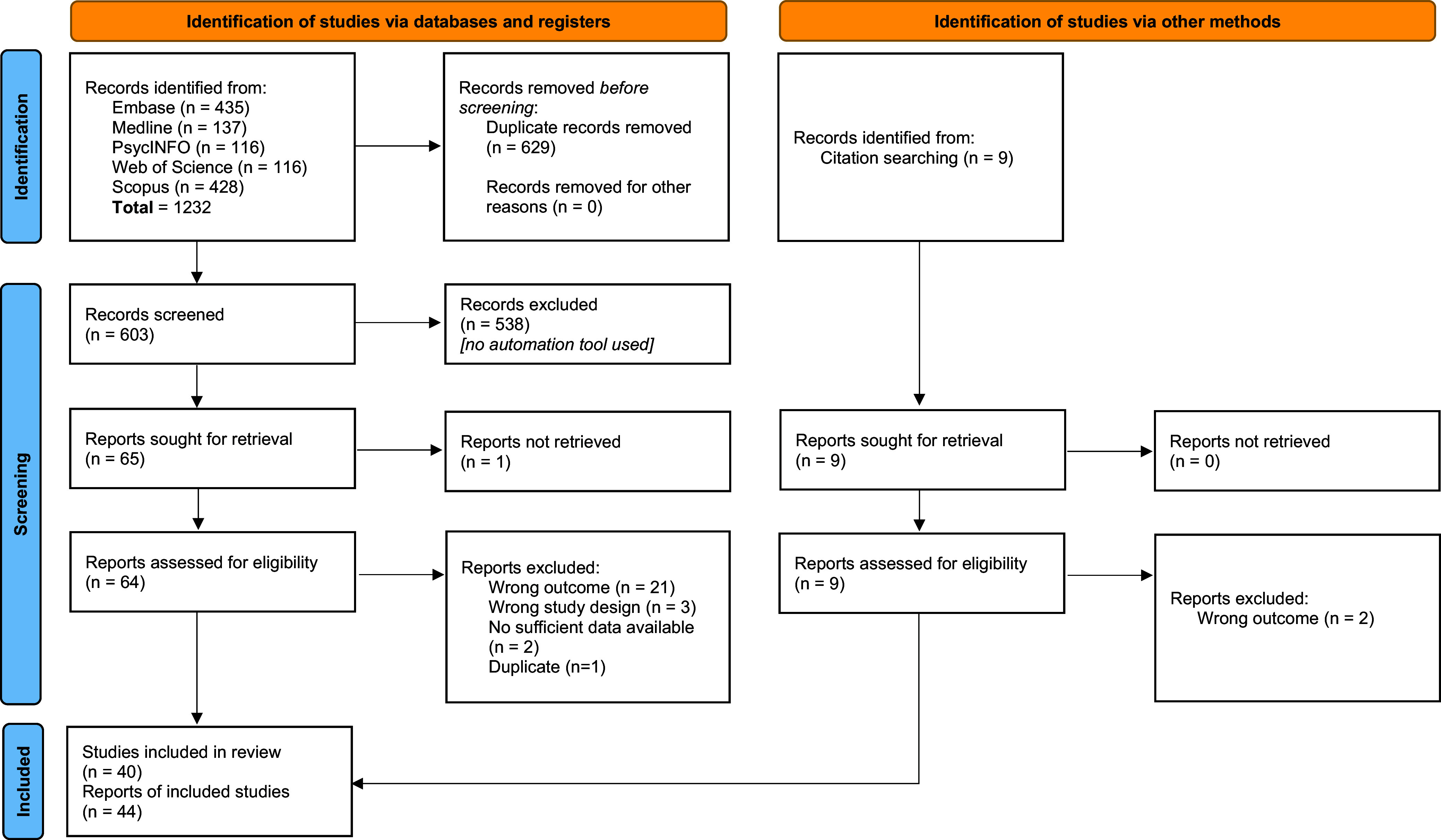
PRISMA 2020 flow diagram for study selection. *From:* Page et al. (2021). For more information, visit: http://www.prisma-statement.org/.

Figure 1 shows the PRISMA flowchart. The database search returned 1232 articles. After removing duplicates, 603 articles were identified. Abstract screening detected 65 potentially eligible papers. On review of the full texts, 37 were deemed eligible for systematic review, and seven additional eligible papers were identified via handsearching references. In total, 44 studies met systematic review inclusion criteria.

### Study characteristics

Table 1 summarizes study characteristics. Thirty-seven articles were retrospective studies, six were case-control studies and one was prospective. Of these, four were pharmacovigilance studies investigating blood dyscrasia within spontaneous reports of clozapine-related side effects. Three papers focused on clozapine rechallenge after an episode of neutropenia or leukopenia warranting clozapine interruption.

**Table 1.** Study characteristics

Author	Country	Study design	Sample size	Clozapine treatment duration (mean days)	Blood dyscrasia (BD)	Definition of BD	n BD	Characteristics	Adjusted confounders	NOS
Hummer et al. 1992 ^a^	Austria	Retrospective	68	119	Neutropenia (transient)	ANC <2000/mm^3^, which returned to normal with no clozapine dose change	8	Age Gender Time to event	None	Poor
Lieberman & Alvir, 1992 ^b^	USA	Retrospective	11382	Not specified	Agranulocytosis	ANC <500/mm^3^	73	Age Gender	None	Good
Alvir, 1993 ^b^	USA	Retrospective	11382	Not specified	Agranulocytosis	ANC <500/mm^3^	73	Age Gender Max clozapine dose Prodrome of agranulocytosis Time to event Baseline WCC	Gender adjusted for age	Good
Banov et al., 1993	USA	Retrospective	118	Follow up <=1 year	Eosinophilia	Eosinophil > 0.7 x 109/L	17	Gender Diagnosis Baseline WCC Baseline Neutrophil count	None	Poor
Alvir & Lieberman, 1994 ^b^	USA	Retrospective	11555	Not specified	Agranulocytosis	ANC <500/mm^3^	73	WCC spike of at least 15% above the previous measurement predicting the occurrence of agranulocytosis within 75 days	None	Good
Hummer et al., 1994 ^a^	Austria	Retrospective	68	117	Neutropenia Eosinophilia Leukocytosis Leukopenia	ANC <2000/mm^3^ Eosinophils> 4% of the WCC Leukocytes >10000/mm^3^ Leukocytes <3500/mm^3^	8 29 28 11	Clozapine plasma level Concomitant medication	None	Poor
Alvir et al., 1995 ^b^	USA	Retrospective	11555	Not specified	Agranulocytosis	ANC <500/mm^3^	73	WCC spike of at least 15% above the previous measurement predicting the occurrence of agranulocytosis within 75 days	None	Good
Atkin et al., 1996	UK	Retrospective	6316	Not specified	Agranulocytosis Neutropenia	ANC <500/mm^3^ ANC 500–1500/mm^3^	48 182	Gender Ethnicity Clozapine dose Time to event	None	Poor
Mauri et al., 1998	Italy	Prospective	16	63	Neutrophil and leukocyte count	Not specified	not specified	Age Gender Clozapine dose Duration of illness Clozapine plasma levels Norclozapine plasma levels Norclozapine/clozapine plasma level ratio	None	Poor
Copolov et al., 1998	Australia	Retrospective	4061	Not specified	Neutropenia Agranulocytosis	ANC 500–1500/mm^3^ ANC <500/mm^3^	60 37	Age Gender Time to event (first 18 weeks) Neutrophil spike before agranulocytosis	None	Poor
Munro et al., 1999	UK	Retrospective	12760	Not specified	Neutropenia Agranulocytosis	ANC 500–1500/mm^3^ ANC <500/mm^3^	344 93	Age Ethnicity Clozapine dose Time to event Baseline WCC Baseline Hb	None	Poor
Lambertenghi Deliliers, 2000	Italy	Retrospective	2404	Not specified	Neutropenia Agranulocytosis Leukocytosis Eosinophilia Thrombocytopenia	ANC 500–1500/mm^3^ ANC <500/mm^3^ Leukocytes >15000/mm^3^ Eosinophil > 400/mm^3^ Platelets <100000/mm^3^	22 16 185 52 2	Age Gender Baseline WCC Clozapine dose	None	Poor
Dettling et al., 2001	Germany	Case-control	108	Not specified	Agranulocytosis	ANC <500/mm^3^	31	Age Gender	None	Good
Kang et al., 2006	Korea	Retrospective	6782	Not specified	Neutropenia Agranulocytosis	ANC 500–1500/mm^3^ ANC <500/mm^3^	697 54	Age Gender Time to event Baseline WCC Baseline ANC	None	Poor
Dunk et al., 2006	UK	Retrospective	53	Not specified	Blood dyscrasia on rechallenge: - Neutropenia - Leukopenia - Agranulocytosis	Total ANC 500–1500/mm^3^ Leukocytes <3000/mm^3^ ANC <500/mm^3^	20 2 9 9	Age Gender Time to event Ethnicity	None	Poor
Maher et al., 2013	USA	Retrospective	87	Not specified	Mild neutropenia Moderate neutropenia Severe neutropenia	ANC 1500–2000/mm^3^ ANC 1000– 1500/mm^3^ ANC <1000/mm^3^	27 17 1	Age Gender Ethnicity	None	Poor
Abanmy et al., 2014	Saudi Arabia	Retrospective	147	Not specified	Blood dyscrasias Agranulocytosis Thrombocytopenia Lymphocytopenia Eosinophilia	WBC<3500/mm^3^ & ANC<2000/mm^3^ ANC<1000/mm^3^ & WBC<2000/mm^3^ Platelets <150000/mm^3^ Lymphocytes <1000/mm^3^ Eosinophil > 0.5 x 109/L	61 16 7 19 40	Age Gender Time to event	None	Poor
Balda et al., 2015	Argentina	Retrospective	73831	Not specified	Agranulocytosis Neutropenia Leukopenia	ANC <500/mm^3^ ANC <2000/mm^3^ WBC <3500/mm^3^	38 285 137	Age Gender Time to event Concomitant medication Clozapine dose	None	Good
Lau & Yim, 2015	China	Retrospective	980	Not specified	Neutropenia^a^ Agranulocytosis^a^ *grouped together due to low n	ANC <1500/mm^3^ ANC <500/mm^3^	13 3	Age Gender Time to event Concomitant medication	None	Poor
Meyer et al., 2015	UK	Retrospective	19	Not specified	Unsuccessful rechallange: - Neutropenia - Agranulocytosis	ANC <1500/mm^3^ ANC <500/mm^3^	2 2	Age Gender Ethnicity ANC nadir on first exposure Time to event at first blood dyscrasia Duration of clozapine break before rechallange Valproate treatment	None	Poor
Demler et al., 2016	USA	Retrospective	193	Not specified	Leukopenia AND /OR Neutropenia Non rechallengeable	WBC<3500/mm^3^ &/OR ANC<2000/mm^3^ WBC<3000/mm^3^ (BEN<3000/mm^3^) &/OR ANC<1500/mm^3^ (BEN<1000/mm^3^)	1148 16	Age Gender Ethnicity Monitoring frequency	None	Poor
Yağcıoğlu 2016	Turkey	Case-control	101	Agranulocytosis cases: median 144 months Controls: median 6 months	Agranulocytosis	ANC <500/mm^3^	10	Age Gender Time to event Clozapine dose Age at illness onset Duration of illness Age at clozapine initiation	None	Poor
Prokopez et al., 2016	Argentina	Retrospective	19	Not specified	Blood dyscrasia on rechallenge	WBC<3000/mm^3^ &/OR ANC<1500/mm^3^	6	Age Gender Neutropenia at first exposure Leukopenia at first exposure Break in clozapine treatment Time to event	None	Poor
Fabrazzo et al., 2017	Italy	Retrospective	135	Not specified	Neutropenia Neutrophilia Leukocytosis Leukopenia Anemia Eosinophilia Thrombocytopenia Thrombocytosis Agranulocytosis	ANC 500–1500/mm^3^ ANC >7000/mm^3^ WBC >15000/mm^3^ WBC <3500/mm^3^ RBC <4.5×106/μL&/or Hb< 12 g/dL Eosinophils >300/mm^3^ Platelets <150000/mm^3^ Platelets >500000/mm^3^ ANC <500/mm^3^	6 55 51 6 71 72 3 4 3	Gender Transient vs persistent	None	Poor
Vargas et al., 2017	Chile	Retrospective	60	127 ± 88 weeks	Mild leukopenia &/OR neutropenia Moderate leukopenia &/OR neutr Severe leukopenia &/OR neutr Any leukopenia &/OR neutropenia	Leukocytes 3000–3500/mm^3^ &/OR ANC 1500–2000/mm^3^ Leukocytes 2000–3000/mm^3^ &/OR ANC 1000– 1500/mm^3^ Leukocytes <1000/mm^3^ &/OR ANC <1000/mm^3^ Leukocytes <3500/mm^3^ &/OR ANC <2000/mm^3^	5 1 2 8	Age Gender Clozapine dose Time to event Age at treatment start Baseline WCC Baseline ANC Previous eosinophilia	None	Poor
Hollingworth et al., 2018	Australia	Retrospective	1173	Not specified	Neutropenia (including agranulocytosis)	Not specified	1173	Gender	None	Poor
Malik et al., 2018	UK	Case-control	272	Controls: >= 3 months of clozapine prescriptions	Red result (Leukopenia/Neutropenia warranting clozapine cessation)	WCC <3000/mm^3^ AND/OR ANC <1500/mm^3^	136	Age Gender Ethnicity Clozapine dose Concomitant anticonvulsants Concomitant valproate Concomitant lithium Concomitant antidepressants Concomitant SSRIs Concomitant statins	Multivariable analysis was conducted for each variable adjusted for age, ethnicity and concurrent sodium valproate use	Good
Royer et al., 2019	France	Retrospective	77	Not specified	Neutropenia (all grades)	ANC <2000/mm^3^	12	Gender Time to event Clozapine dose Clozapine started as monotherapy (vs polytherapy)	None	Poor
Tunsirimas et al., 2019	Thailand	Retrospective	641	3.82 years (range 0.01–15.58)	Leukopenia Agranulocytosis	WBC <3500/mm^3^ ANC <500/mm^3^	20 0	Age Gender Time to event Clozapine dose Baseline WCC Baseline ANC	None	Poor
Mena et al., 2019	Chile	Retrospective	5380	Median: 382 weeks (IQR: 158–589)	Mild neutropenia Moderate neutropenia Agranulocytosis	ANC 1000–1499/mm^3^ ANC 500–999/mm^3^ ANC <500/mm^3^	209 69 33	Age Gender Time to event Clozapine dose Previous episode of neutropenia	Age groups were adjusted for gender and clozapine dose	Good
Matsui et al., 2020	Japan	Retrospective	3746	Median 1.5 years	Neutropenia / Leukopenia Agranulocytosis	WBC <3000/mm^3^ &/OR ANC <1500/mm^3^ ANC <500/mm^3^	182 38	Age Gender Time to event Clozapine dose Baseline ANC Baseline WCC	None	Good
Toyoda et al., 2021 ^c^	Japan	Retrospective	8263	Not specified	Neutropenia Leukopenia Agranulocytosis	ANC <1500/mm^3^ WBC <3000/mm^3^ ANC <500/mm^3^	NA	Age	None	Poor
Imazu et al., 2021 ^c^	Japan	Retrospective	8264	Not specified	Neutropenia Leukopenia Agranulocytosis	ANC <1500/mm^3^ WBC <3000/mm^3^ ANC <500/mm4	NA	Baseline neutrophil count Baseline leukocyte count	None	Poor
Tsukiji et al., 2021	Japan	Nested case-control	228	Not specified	Eosinophils	Not specified	NA	Use of valproate	Adjusted for gender, age, smoking status, lithium use	Good
Gee & Taylor, 2021	UK	Retrospective	56	Not specified	Reduction from baseline of: - Absolute neutrophil count - Lymphocyte count - WCC	Reduction from baseline to day 0–7 after COVID + test	NA	Covid infection	None	Poor
Johannsen et al., 2022	Denmark	Retrospective *(within-subject design)*	520	773 person-years of clozapine exposure	Neutropenia Neutropenia A Neutropenia B Both Neutropenia A and B	ANC <1500/mm^3^ >=2 consecutive neutrophil counts of <1500/mm^3^ within 7 days A single neutrophil count of < 1500/mm^3^ Any neutrophil count <1500/mm^3^	NA 81 7 7	Age Gender Time to event Baseline WCC Baseline ANC Baseline eosinophils Baseline thrombocytes Baseline lymphocytes Baseline monocytes	None	Good
Glocker et al., 2023	Germany	Retrospective	38349	Not specified	Neutropenia Agranulocytosis	ANC <1500/mm^3^ ANC <500/mm^3^	67 41	Clozapine dose	None	Poor
Yang et al., 2023	Taiwan	Retrospective	1084	>= 3 years of follow up	Neutropenia Leucocytosis	WBC counts < 3000/mm^3^ OR ANC < 1500/mm^3^ WBC > 11000/mm^3^	48 453	Age Gender Clozapine dose Concomitant valproate Concomitant lithium Dose of concomitant lithium Concomitant valproate + lithium Concomitant benzodiazepine	Adjusted for age, gender, haematological events, clozapine dose, concomitant psychotropics	Good
Uwai & Nabekura, 2023	Japan	Retrospective	2453	Not specified	Leukopenia Neutropenia Agranulocytosis	Not specified	143 195 118	Concomitant lithium	Adjusted OR, covariates unclear	Poor
Kang et al., 2023	Korea	Retrospective	1408	Not specified	Neutropenia Agranulocytosis	Not specified	189 4	Fever	None	Poor
Northwood et al., 2024	Australia and AotearoaNew Zealand	Retrospective	26630	Median 195 weeks (IQR 36–525)	Isolated Mild Neutropenia Serious neutropenia leading to clozapine cessation Serious neutropenia unrelated to clozapine	ANC 1000–1500/mm^3^ ANC < 1000/mm^3^ leading to clozapine cessation ANC < 1000/mm^3^ unrelated to clozapine	1146 313 223	Age Gender Time to event First absolute neutrophil count (<2500 /mm^3^ vs ≥2500/mm^3^) Previous drug-induced neutropenia Benign ethnic neutropenia or other need for special range	None	Poor
Rubio et al., 2024	Finland	Case-control	14037	Median 398 (IQR 95–1193)	Agranulocytosis	Not specified	231	Concomitant non-clozapine antipsychotic	Clozapine dose, medical comorbidities and concomitant treatments	Good
Bleich et al., 2024	Germany	Retrospective	38349	Not specified	Blood dyscrasia Neutropenia Agranulocytosis Eosinophilia	Any blood count changes ANC 500–1500/mm^3^ ANC < 500/mm^3^ Eosinophils >1500/mm^3^	Not specified	Age Gender Polipharmacy Clozapine dose	None	Poor
Kikuchi et al., 2024	Japan	Case-control	241	Follow up of 90 days	Eosinophilia	Eosinophils >600/mm^3^	48	Age Gender BMI Fast vs slow titration Concomitant Valproate Smoking status” Inflammatory Adverse Events (severe and non-severe)	Clozapine titration speed and concomitant valproic acid use	Good

*Note*: ANC, absolute neutrophil count; NOS, Newcastle-Ottawa Scale; WBC, white blood cells.

a Same sample size.

b Same sample size.

c Same sample size.

Study sample sizes ranged from 19 to 73,831 participants, and included cohorts across Europe, North America, South America, Asia, and Oceania with a total of 285,658 individuals. Table 1 expands on study characteristics of the papers included in the systematic review.

Twenty-three studies focused on neutropenia (excluding agranulocytosis), 24 studies identified instances of agranulocytosis, five reported a combined outcome of neutropenia or agranulocytosis, and two investigated changes in neutrophil counts. Eight papers gave information on episodes of leukopenia, whilst six studies did not distinguish between instances of leukopenia and neutropenia, grouping them together as “red results”. Additionally, eight papers gave information on episodes of eosinophilia, four on leukocytosis, and one on anemia (Table 1).

Potential risk factors investigated included: gender, age, clozapine dose or plasma concentration during blood dyscrasia, baseline white cell counts (WCC), concomitant psychotropic medication, neutrophil or leucocyte spike before episode of agranulocytosis, duration of illness, duration of clozapine break before rechallenging, fever, and transient vs. persistent nature of blood dyscrasia. Twenty studies reported information on length on clozapine treatment before blood dyscrasia.

### Risk factors

#### Gender

##### Agranulocytosis

No significant gender difference in risk of agranulocytosis was found in 8 studies (*n* = 50,534) (Atkin et al., 1996; Copolov et al., 1998; Dettling et al., 2001; Kang et al., 2006; Lambertenghi Deliliers, 2000; Matsui et al., 2020; Mena et al., 2019; Yagcioglu et al., 2016a), whilst two papers (*n* = 85,213) found higher incidence of agranulocytosis in females (Alvir et al., 1993; Balda et al., 2015).

##### Other blood dyscrasia

Most papers did not find any association between gender and risk of neutropenia during clozapine treatment (*n* = 14,636) (Atkin et al., 1996; Johannsen et al., 2022; Lambertenghi Deliliers, 2000; Mauri et al., 1998; Mena et al., 2019). One study (*n* = 87) found increased risk of neutropenia in male patients (Maher et al., 2013); and two in female patients (*n* = 1250) (Hollingworth et al., 2018; Royer et al., 2019), one of which was an Australian pharmacovigilance study that specified an OR of 1.45 (95% CI: 1.28–1.67). Another recent pharmacovigilance study (*n* = 26,630) (Northwood et al., 2024) reported increased risks in females for episodes of minor neutropenia (OR 1.23, 95% CI: 1.05–1.45), and serious neutropenia leading to clozapine discontinuation (OR 1.37, 95% CI: 1.04–1.79), whilst no gender difference was observed for serious neutropenia that did not result in clozapine cessation (OR 1.28, 95% CI: 0.82–2.04).

Four studies found no association between gender and increased risk of blood dyscrasias (leukopenia and neutropenia combined) (*n* = 1127) (Abanmy et al., 2014; Lau & Yim, 2015). Similarly, no gender difference was reported for red results (*n* =272) (Malik et al., 2018). In contrast, two studies identified an increased risk among female patients for general changes in blood count (*n* = 38,349) (Bleich et al., 2024), and leukopenia (OR: 2.78, 95% CI: 1.14–7.14).

Demler et al. (2016) found that during clozapine rechallenge men were more likely to have blood dyscrasias and less likely to have no events at all (*n* = 193), whilst Prokopez found no significant difference in blood dyscrasia on rechallenge between men and women (*n* = 19) (Prokopez et al., 2016).

With regard to eosinophilia, one study found an increased cumulative incidence in women (*n* = 118) (Banov et al., 1993), one in men (*n* = 147) (Abanmy et al., 2014), and one found no gender difference (*n* = 241) (Kikuchi et al., 2024).

One study reported an increased incidence of leukocytosis in women (*n* = 1084) (Yang et al., 2023).

#### Age

##### Agranulocytosis

Some studies reported an increased risk of agranulocytosis with increased age (*n* = 17,497) (Dettling et al., 2007; Matsui et al., 2020; Mena et al., 2019; Toyoda et al., 2021). More specifically, in the same cohort (*n* = 11,382) Lieberman and Alvir (1992) and Alvir et al. (1993) found an increase of about 6% (95% CI: = 5 to 8%, *p* < 0.001) with each year of age, although risk was also found higher in those <21 years old. Atkin et al. (1996) found a marginal increase in incidence of agranulocytosis with older age (in particular for >50 years); Copolov et al. (1998) (*n* = 4061) found that patients who developed agranulocytosis were significantly older than those without agranulocytosis. Munro et al. (1999) (*n* = 12,760) reported an increase in risk of agranulocytosis by 53% for each 10-year increase in age and highlighted that adolescents have the lowest risk of agranulocytosis. Balda et al. found an increased risk of agranulocytosis with older age (OR 1.22, 95% CI: 1.02–1.45). Four studies found no significant difference in age between those who developed agranulocytosis and those who didn’t (*n* = 45,379) (Abanmy et al., 2014; Bleich et al., 2024; Kang et al., 2006; Yagcioglu et al., 2016a).

##### Other blood dyscrasia

No age difference was found in seven papers analyzing neutropenia when excluding agranulocytosis cases (*n* = 87,605) (Atkin et al., 1996; Bleich et al., 2024; Copolov et al., 1998; Kang et al., 2006; Maher et al., 2013; Mena et al., 2019; Northwood et al., 2024); whilst one study found that older age was associated with increased likelihood of developing neutropenia during clozapine treatment (*n* = 68) (Hummer et al., 1992). Johannsen et al (*n* = 520) (Johannsen et al., 2022) found no age difference in risk of having one or more episodes of neutrophils <1500/mm^3^ during periods on versus off clozapine in a within-subject design study.

With regard to reduced neutrophil and leukocyte count grouped together, no difference in age was found in six papers (*n* = 12,555) (Abanmy et al., 2014; Lambertenghi Deliliers, 2000; Mauri et al., 1998; Toyoda et al., 2021; Tunsirimas et al., 2019; Yang et al., 2023); whilst one paper found an increased risk for patients aged 40–59, as opposed to patients between the ages of 20–29 and 60–69 who were more likely to be event free (*n* = 193) (Demler et al., 2016). Malik et al. (2018) (*n* = 272) found that the risk of red results on clozapine was higher in younger patients, with odds for those aged 17–29 years 6 times greater than those aged >=60 (OR 6.34, *p* = 0.001).

Older age at the time of clozapine rechallenge was associated with unsuccessful rechallenge in one study (*n* = 19) (Meyer et al., 2015), whilst no association was found in another study (*n* = 19) (Prokopez et al., 2016).

#### Clozapine dose and plasma concentration

##### Agranulocytosis

No association with risk of agranulocytosis was found in six studies (*n* = 27,002) (Alvir et al., 1993; Atkin et al., 1996; Matsui et al., 2020; Mena et al., 2019; Royer et al., 2019; Yagcioglu et al., 2016a), whilst a reduced risk at higher doses was found by Munro et al. (1999) (*n* = 12,760) (HR 0.787, 95% CI: 0.702–0.882).

##### Other blood dyscrasia

Clozapine dose was not associated with risk of neutropenic events (excluding agranulocytosis) in three studies (*n* = 44,742) (Atkin et al., 1996; Glocker et al., 2023; Royer et al., 2019). Munro et al. (1999) (*n* = 12,760) reported a risk reduction of 31% for each 100 mg increase in clozapine dose.

Plasma concentration of clozapine was not associate with increased risk of developing blood dyscrasia in two studies (*n* = 2472) (Hummer et al., 1994; Lambertenghi Deliliers, 2000); whilst Malik et al. (2018) (*n* = 272) concluded that patients who had a red result received lower doses of clozapine than those who didn’t. No association with episodes of leukopenia was found in one study (*n* = 641) (Tunsirimas et al., 2019), whilst negative association was found with regard to neutropenia and leukopenia grouped together by Matsui et al (*n* = 3746) (Matsui et al., 2020). Leukocytosis correlated with clozapine dose in a study investigating effect of lithium during clozapine treatment (*n* = 1084) (Yang et al., 2023), with an odds ratio of 1.11 (95% CI: 1.02–1.22).

Mauri et al. (1998) (*n* = 16) found a positive correlation between clozapine and norclozapine plasma concentration and neutrophil count, and a negative correlation between norclozapine/clozapine ratio (considered an index of clozapine metabolism) and neutrophil count.

#### Baseline cell counts

##### Agranulocytosis

Alvir et al. (1993) (*n* = 11,382) reported that baseline WCC was inversely related to risk of agranulocytosis, but the association was small (RR 0.988, 95% CI: 0.978–0.998) and not significant after adjustment for gender and age.

##### Other blood dyscrasia

Munro et al. (1999) (*n* = 12,760) reported that for every 100/mm^3^ lower baseline WCC, the hazard of subsequently meeting the threshold for neutropenia (excluding agranulocytosis) increased by 31%. Among those who developed neutropenia on clozapine, both Caucasian and Afro-Caribbean patients had lower initial WCCs compared to those who did not. Similarly, Kang et al. (2006) found that pre-treatment WCC and neutrophil counts were significantly lower in patients who developed neutropenia than in those who developed agranulocytosis or experienced no blood dyscrasia. Johannsen et al (*n* = 520) (Johannsen et al., 2022) identified a baseline neutrophil count <2000/mm^3^ as a strong predictor of clozapine-associated neutropenia (OR 24.4, 95% CI: 6.4–215.0). Northwood et al (*n* = 26,630) (Northwood et al., 2024) also found that a baseline neutrophil count <2500/mm^3^ was associated with increased odds of developing minor neutropenia on clozapine.

Two studies (*n* = 3045) (Lambertenghi Deliliers, 2000; Tunsirimas et al., 2019) did not find any association between baseline WCC and leukopenia or any neutropenia on clozapine; whilst three studies found significantly lower baseline WCC and neutrophil count in those who developed leukopenia and/or neutropenia during clozapine treatment (*n* = 12,070) (Imazu et al., 2021; Matsui et al., 2020; Vargas et al., 2017).

Banov et al. (1993) (*n* = 118) reported no significant change between WCC and neutrophil count at baseline and the week of peak eosinophil count in eosinophilia cases.

#### Ethnicity

##### Agranulocytosis

No significant association between ethnicity and agranulocytosis was reported in one study (*n* = 6316) (Atkin et al., 1996). Another study (*n* = 12,760) found that, compared to Caucasians, individuals of Asian ethnicity had a 2.4-fold increased risk of agranulocytosis, while risks among Oriental/mixed race and Afro-Caribbean groups were not significantly different (*p* = 0.84; *p* = 0.61, respectively) (Munro et al., 1999).

##### Other blood dyscrasia

African and Afro-Caribbean ethnicity was found to be significantly more likely than other ethnic groups to have pre-treatment neutropenia (2.8% vs. 0.2%, *n* = 6316) (Atkin et al., 1996) and to develop neutropenia on clozapine compared to Caucasians (HR 1.77, 95% CI: 1.21–2.58, *n* = 12,760) (Munro et al., 1999). Of note, these findings did not account for benign ethnic neutropenia (BEN), which may act as a confounding factor. However, one study found no significant difference in neutropenia risk between Caucasians and African Americans (*n* = 87) (Maher et al., 2013).

When leukopenia and neutropenia were combined, one study reported more overall events in Caucasians, but moderate episodes were more frequent among African Americans (*n* = 193) (Demler et al., 2016). Malik et al. (2018) (*n* = 272) found that individuals of Black ethnicity had nearly three times the odds of red results compared to those of White or Asian ethnicity (OR 2.99, 95% CI: 1.74–5.12, multivariate analysis).

#### Concomitant psychotropic medication

##### Agranulocytosis

Several studies identified increased risk of neutropenia or agranulocytosis with concomitant psychotropic medication. Balda et al. (2015) (*n* = 73,831) found higher odds of agranulocytosis in patients prescribed additional antipsychotics compared to clozapine monotherapy (OR 2.22, 95% CI: 1.09–4.54). Rubio et al. (2024) (*n* = 114,037) similarly reported increased risk with concurrent non-clozapine antipsychotics (OR 1.59, 95% CI: 1.11–2.27) and high-dose antipsychotic regimens (OR 1.34, 95% CI: 1.01–1.78). Lau and Yim (2015) (*n* = 980) observed that 14 out of 16 patients who developed neutropenia or agranulocytosis were receiving other psychotropics, most commonly anticonvulsants, particularly valproate. Conversely, Bleich et al. (2024) (*n* = 38,349) reported a higher incidence of agranulocytosis with clozapine alone (8.8%) than in combination with other antipsychotics (3.2%, *p* = 0.012).

##### Other blood dyscrasia

Valproate use was associated with increased risk of neutropenia in two studies (*n* = 4830) (Matsui et al., 2020; Yang et al., 2023). Malik et al. (2018) (*n* = 272) found a dose-dependent relationship, with adjusted odds of neutropenia rising significantly at doses ≥1000 mg/day (OR 5.59 and OR 13.2, respectively). However, Yang et al. (2023) (*n* = 1084) did not replicate this dose-response trend (*p* = 0.808). In the same study, Malik et al. found that antidepressant use – particularly SSRIs – was protective against neutropenia (OR 0.44, 95% CI: 0.22–0.91 for all antidepressants; OR 0.33, 95% CI: 0.15–0.75 for SSRIs).

Lithium use was associated with increased odds of leukocytosis during clozapine treatment (OR 3.39, 95% CI: 1.72–6.68, *n* = 1084), with a dose-dependent effect (Yang et al., 2023). Lithium was not associated to increased risk of leukopenia in a pharmacovigilance study by Uwai and Nabekura (2023) (*n* = 2453).

Regarding eosinophilia, one study (*n* = 228) (Tsukiji et al., 2021) found a significant association between concomitant valproate use and the occurrence of eosinophilia four weeks after clozapine initiation, along with elevated baseline eosinophil counts in patients prescribed valproate. In contrast, another study (Kikuchi et al., 2024) found no significant association (OR 0.61, 95% CI: 0.23–1.60, *n* = 241).

Hummer et al. (1994) (*n* = 68) reported no relationship between psychotropic co-medication and either leukopenia or leukocytosis.

#### Neutrophil or leucocyte spike before agranulocytosis

##### Agranulocytosis

Alvir and Lieberman (1994) found that a white cell count (WCC) spike of ≥15% above the previous measurement was associated with an increased risk of agranulocytosis within 75 days (RR 3.02, 95% CI: 1.38–6.57, *n* = 11,555). However, a re-analysis of the same cohort (Alvir et al., 1995), showed that 87% of non-cases also exhibited at least one spike, suggesting poor specificity. Similarly, Copolov et al. (1998) reported that neutrophil spiking had limited predictive value, with a sensitivity of 0.76, specificity of 0.06, and positive predictive value of only 0.01 (*n* = 4061).

##### Other blood dyscrasia

Meyer et al. (2015) (*n* = 19) found that three out of four individuals who developed neutropenia during a clozapine rechallenge showed a spike in ANC within the two weeks preceding the dyscrasia.

#### Duration of illness

No association between duration of illness and any blood dyscrasia was found in two studies (*n* = 117) (Mauri et al., 1998; Yagcioglu et al., 2016a).

#### Fever and inflammatory events

Kang et al. (2023) reported that patients who developed fever during clozapine titration had a higher incidence of neutropenia compared to those without fever (9.0% vs. 4.6%; χ^2^=8.45, *p* = 0.004, *n* = 1408), while the incidence of agranulocytosis did not significantly differ between the two groups (1.0% vs. 0.1%; χ^2^ = 5.37, *p* = 0.050). Kikuchi et al. (2024) (*n* = 241) found that the risk of eosinophilia increased with the severity of clozapine-related inflammatory adverse events, which typically preceded its onset. Multivariate analysis revealed higher odds of eosinophilia in patients with severe (OR 28.4, 95% CI: 6.90–116) versus non-severe inflammation (OR 6.15, 95% CI: 2.80–13.5), supporting the hypothesis that eosinophilia is driven by clozapine-induced inflammation.

#### Length of clozapine treatment

##### Agranulocytosis

Ten studies reported that the majority of agranulocytosis cases occurred within the first weeks of clozapine treatment, with 62.5% to 89.6% of cases arising within 18 weeks of treatment initiation (*n* = 134,773) (Abanmy et al., 2014; Alvir et al., 1993; Atkin et al., 1996; Balda et al., 2015; Copolov et al., 1998; Kang et al., 2006; Mena et al., 2019; Munro et al., 1999; Royer et al., 2019; Rubio et al., 2024). Atkin et al. (1996) observed a tenfold reduction in risk during the second year of treatment, from 0.7% to 0.07% (*p* < 0.05), noting that the risk in year 2 was comparable to the risk associated with phenothiazines. Rubio et al. (2024) found that the adjusted ORs for agranulocytosis decreased over time, from 36.01 (95% CI: 16.79–77.22) in individuals treated for less than six months to 4.38 (95% CI: 1.86–10.34) in those treated for 54 months or longer.

##### Other blood dyscrasia

Regarding neutropenia, approximately 50% of cases occurred within 18 weeks of starting clozapine (*n* = 9224) (Atkin et al., 1996; Johannsen et al., 2022; Kang et al., 2006; Lau & Yim, 2015) or earlier (*n* = 9441) (Copolov et al., 1998; Mena et al., 2019). Atkin et al. (1996) reported that only half of the cases of neutropenia occurred after 18 weeks of treatment, with incidence significantly decreasing from 2.3% in the first year to between 0.5% and 0.7% during years two to four (*p* < 0.005).

In a study on clozapine rechallenge, Prokopez et al. (2016) (*n* = 19) found a shorter median latency to the first blood dyscrasia in patients who did not develop a second event compared to those who did (54 vs. 182 weeks).

Fabrazzo et al. (2017) (*n* = 135) reported that persistent blood dyscrasias appeared sooner than transient ones within the first 18 weeks of treatment. No major change over time was found by Abanmy et al. (2014) (*n* = 147) with regard to thrombocytopenia, lymphocytopenia, and eosinophilia.

### Meta-analysis of the results

Among the 44 studies included, 15 contributed to at least one meta-analysis. Seven risk factors were eligible for meta-analysis based on the consistency of definitions and measurements across studies.

#### Risk factors for neutropenia defined as ANC < 2000/mm^3^

**Figure 2.**
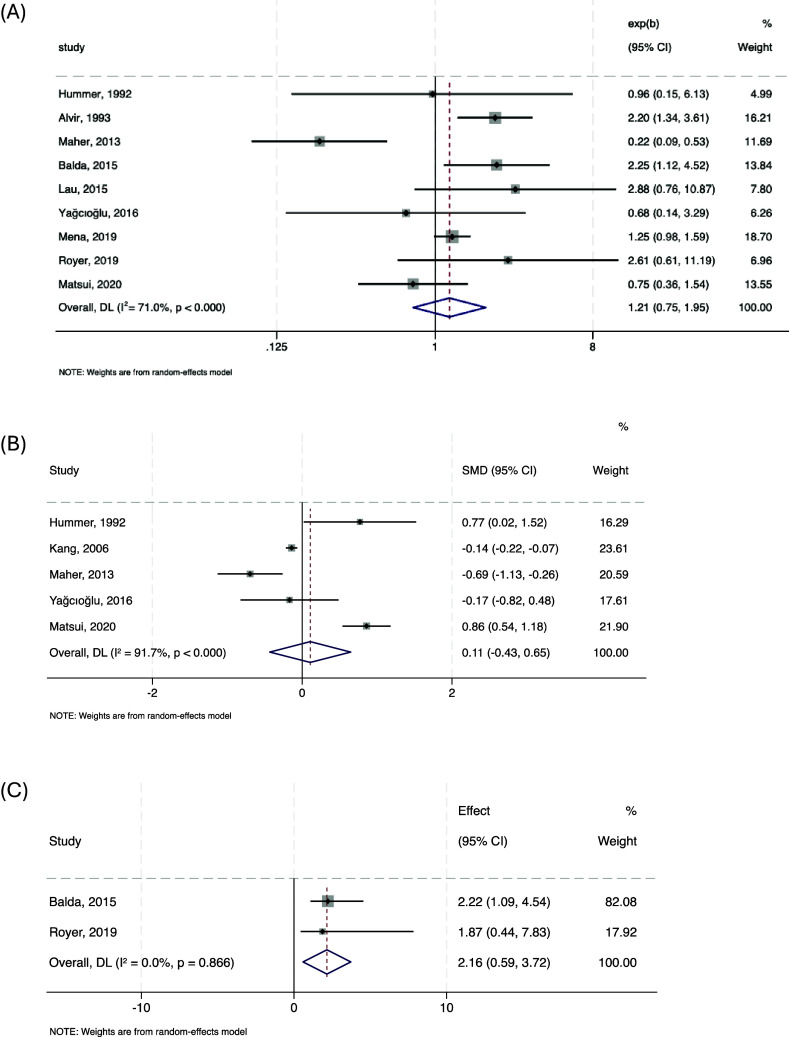
Forest plots showing the association between neutrophils <2000/mm^3^ and female gender (a), age (b), and concomitant medication (c).

Figure 2 shows forest plots of the three meta-analyses examining potential risk factors for clozapine-associated neutropenia, defined as an ANC < 2000/mm^3^. Gender was examined across nine studies (*n* = 21,864), yielding a pooled OR for females of 1.207 (95% CI: 0.748–1.946, *p* = 0.441; *I* ^2^ = 71.0%), indicating no statistically significant association. Age was assessed in five studies (*n* = 11,535), showing no significant effect (pooled SMD = 0.11, 95% CI: –0.43 to 0.65, *p* 0.696; *I* ^2^ = 91.7%). Concomitant medication use was analyzed in two studies (*n* = 73,909), revealing a significant association with neutropenia (pooled OR 2.146, 95% CI: 1.13–4.07, *p* = 0.019; *I* ^2^ = 0%).

#### Risk factors for neutropenia defined as ANC < 1500/mm^3^

**Figure 3.**
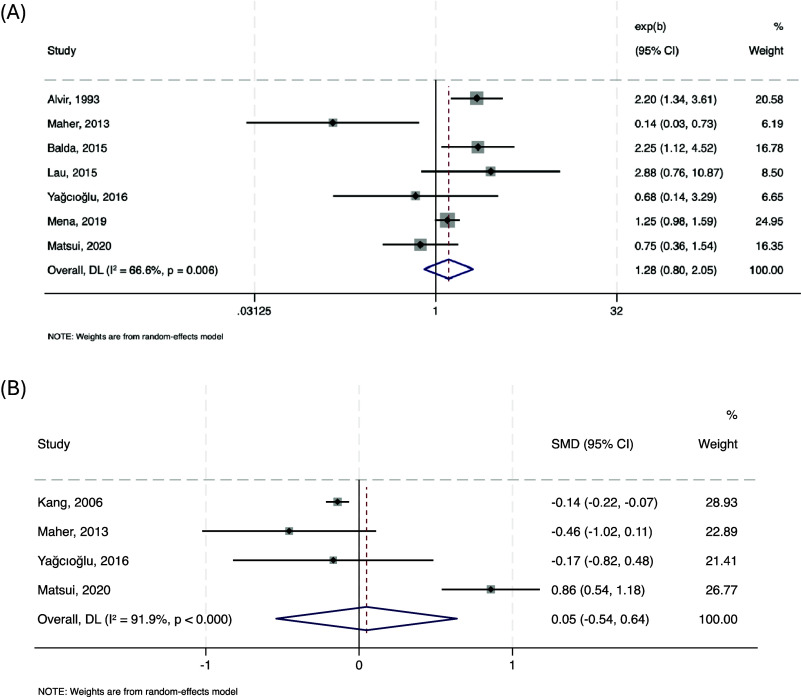
Forest plots showing the association between neutrophils <1500/mm^3^ and female gender (a), and age (b).

No significant association was identified between ANC <1500/mm^3^ and female gender across seven studies (*n* = 95,480; pooled OR = 1.283, 95% CI: 0.803–2.051, *p* = 0.297; *I* ^2^ = 66.6%). Similarly, age was not significantly associated with neutropenia in four studies (*n* = 11,440; pooled SMD = 0.05, 95% CI: –0.54 to 0.64, *p* = 0.872; *I* ^2^ = 91.9%). Forest plots are presented in Figure 3.

#### Risk factors for agranulocytosis defined as ANC < 500/mm^3^

**Figure 4.**
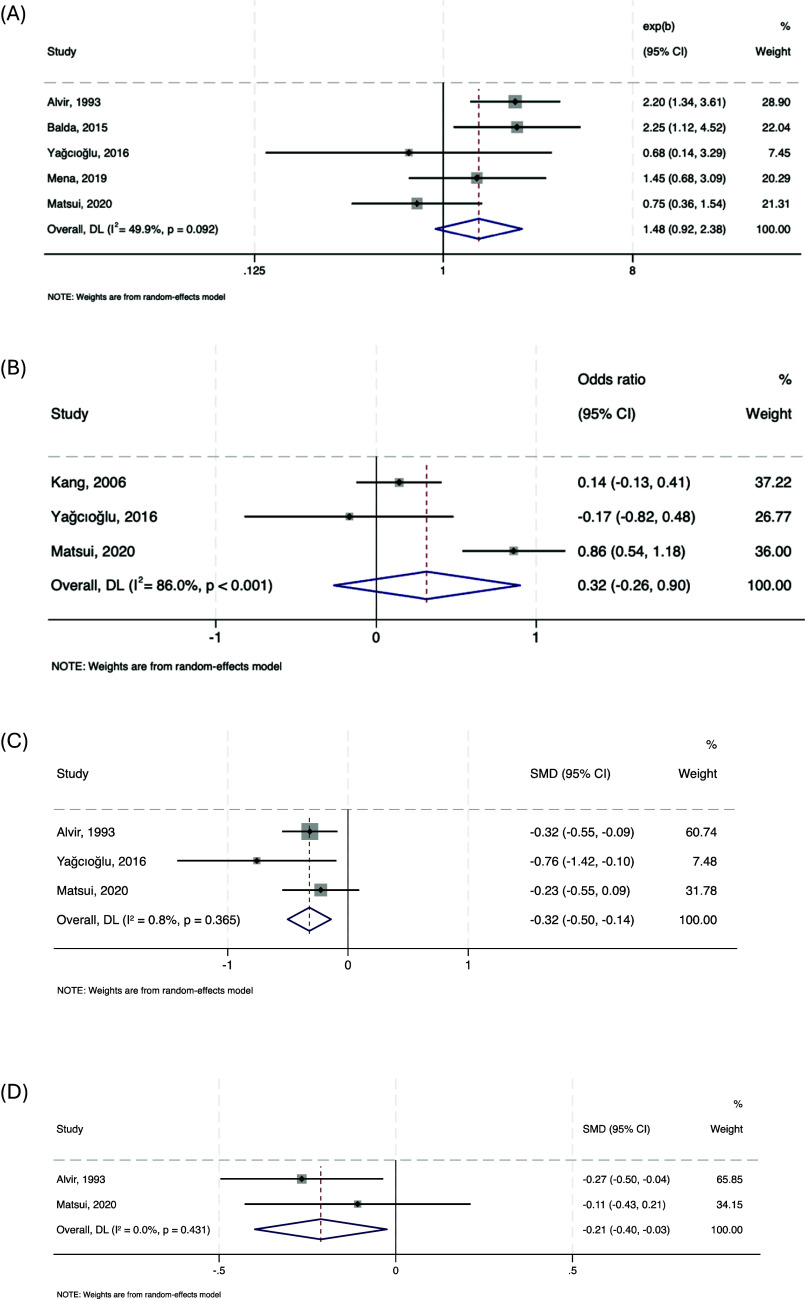
Forest plots showing the association between neutrophils <500/mm^3^ and female gender (a), age (b), clozapine dose (c), and baseline white blood cell count (d).

Meta-analyses were conducted on associations with gender, age, clozapine dose, and baseline WCC (Figure 4). No significant association with female gender was found across five studies (*n* = 94,440; pooled OR = 1.479, 95% CI: 0.921–2.376; *p* = 0.106; *I* ^2^ = 49.9%). Age was examined in three studies (*n* = 10,629), with no significant difference between those who did and did not develop severe neutropenia (pooled SMD = 0.32, 95% CI: –0.26 to 0.90, *p* = 0.285; *I* ^2^ = 86%). A significant association with lower clozapine doses in those who developed severe neutropenia was observed across three studies (*n* = 15,302; pooled SMD = –0.32, 95% CI: –0.50 to –0.14, *p* < 0.001; *I* ^2^ = 0.8%). Baseline WCC showed a significant negative association in two studies (*n* = 15,201; pooled SMD = –0.21, 95% CI: –0.40 to –0.03, *p* = 0.026; *I* ^2^ = 0.0%).

#### Risk factors for blood dyscrasia on clozapine rechallenge

**Figure 5.**
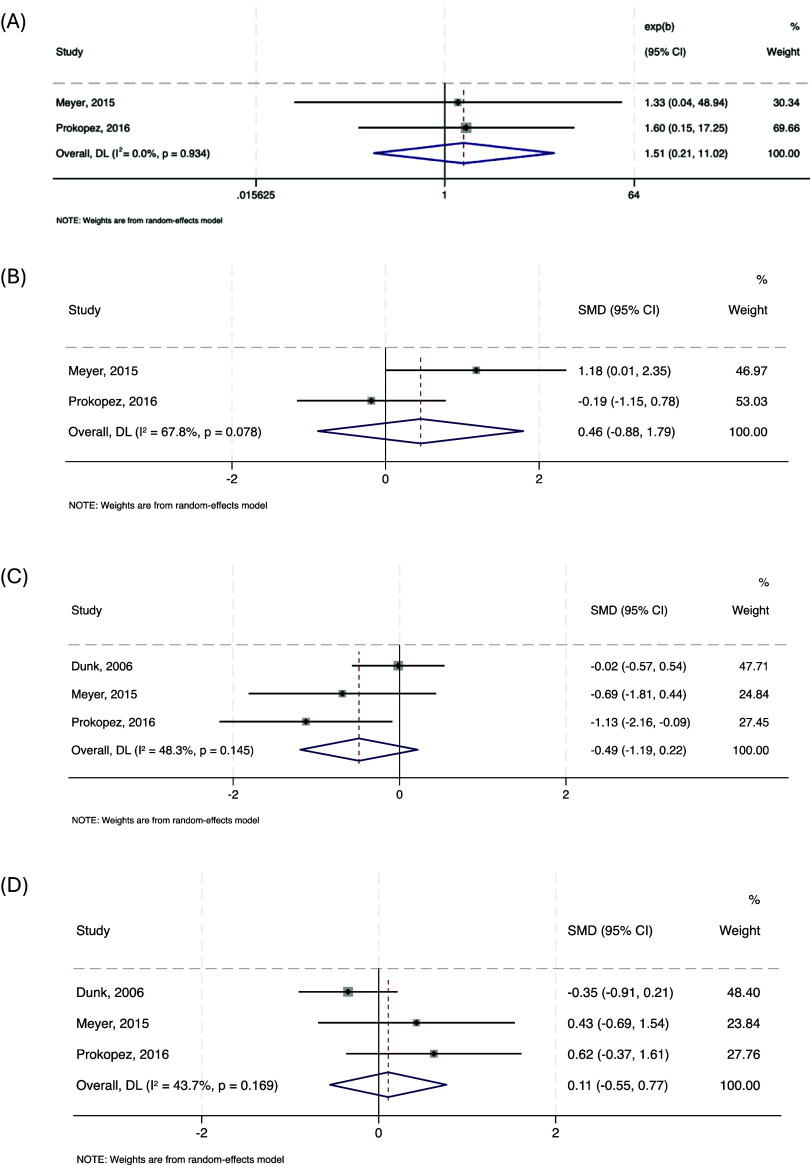
Forest plots showing the association between blood dyscrasia on clozapine rechallenge and female gender (a), age (b), length of clozapine trial (weeks) (c), and length of break before clozapine rechallenge (weeks) (d).

Meta-analysis of two studies (*n* = 38) found no significant association between subsequent blood dyscrasia risk after clozapine rechallenge and either female gender (pooled OR = 1.514, 95% CI: –0.208 to 11.016, *p* = 0.682; *I* ^2^ = 0.0%) or age (pooled SMD = 0.46, 95% CI: –0.88 to 1.79, *p* = 0.503; *I* ^2^ = 67.8%). In a separate meta-analysis of three studies (*n* = 91), neither the duration of the initial clozapine trial (pooled SMD = –0.49, 95% CI: –1.19 to 0.22, *p* = 0.176; *I* ^2^ = 48.3%) nor the length of the discontinuation period prior to rechallenge (pooled SMD = 0.11, 95% CI: –0.55 to 0.77, *p* = 0.749; *I* ^2^ = 43.7%) was significantly associated with the risk of a subsequent blood dyscrasia episode. Forest plots are presented in Figure 5.

#### Risk factors for eosinophilia

**Figure 6.**
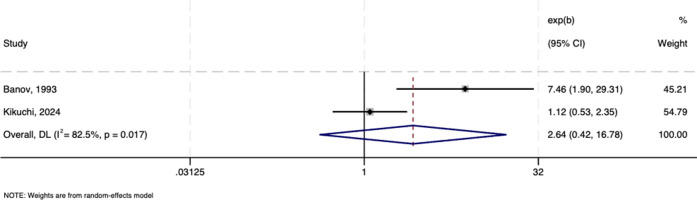
Forest plots showing the association between eosinophilia and female gender.

Figure 6 shows no association between female gender and risk of eosinophilia in a meta-analysis of two studies (*n* = 359; pooled OR = 2.640, 95% CI: 0.415–16.784, *p* = 0.304; *I* ^2^ = 82.5%).

#### Sensitivity analyses

All sensitivity analyses including studies rated as good quality only, yielded results consistent with the primary meta-analyses, suggesting that our results were robust and not substantially influenced by study quality.

#### Publication bias

Funnel plots for all meta-analyses appeared symmetrical, suggesting no statistically significant evidence of small-study effects. However, the relatively small number of studies included in each analysis limits the reliability of funnel plot–based assessments, and the possibility of undetected publication bias cannot be excluded.

## Discussion

In this study, we systematically summarized and synthetized available evidence on risk factors of blood dyscrasia occurring during clozapine treatment, with a focus on neutropenia and agranulocytosis. Despite extensive research and pharmacovigilance efforts, no strong or consistent predictors have been identified, reflecting the complexity and likely multifactorial nature of clozapine-associated hematological events.

A clear and consistent causal association between clozapine and neutropenia has not been definitively established. Some authors have proposed that agranulocytosis represents the only true clozapine-induced hematological side effect (Johannsen et al., 2022; Taylor et al., 2022), supported by recent genetic findings (Konte et al., 2021), but this remains an area of ongoing debate and investigation. For this reason, we placed primary emphasis on agranulocytosis, the most clinically meaningful blood dyscrasia, while grouping less severe hematological abnormalities, including neutropenia, under the broader category of blood dyscrasias.

### Agranulocytosis

Evidence on demographic risk factors has been inconsistent. Our meta-analyses did not identify significant gender- or age-related associations with agranulocytosis, suggesting that these factors do not meaningfully influence risk.

Baseline hematological parameters may appear more relevant. Our analysis of two studies found a negative association between baseline WCC and agranulocytosis risk on clozapine. However, this may reflect physiological inter-individual variability in neutrophil count, with those at the lower end of the normal range being more likely to cross monitoring thresholds. Furthermore, a common cause of low ANC is BEN, a condition in which individuals, typically of certain ethnic backgrounds, have chronically lower ANC without an increased risk of infection or underlying pathology. In some regions, a diagnosis of BEN allows lower ANC thresholds for clozapine treatment to be applied. However, most studies did not assess or exclude BEN, limiting interpretation. Differentiating BEN from pathological vulnerability is crucial to avoid unnecessary exclusion from, or discontinuation of, clozapine treatment. There may also be a case to be made for individualized thresholds based on the baseline value, but this would be complex to administer. A simpler alternative is to extend BEN-adjusted thresholds to anyone with a persistently low baseline ANC, especially where this can be demonstrated to be consistent across time when off clozapine.

The under-diagnosis of BEN also complicates the interpretation of the ethnicity data. One study investigating agranulocytosis found no association with ethnicity, supporting the view that BEN does not lead to increased risk for agranulocytosis. This is further supported by Oloyede et al. (2024) who reported a higher risk of clozapine-induced agranulocytosis in White compared to Black individuals in patients who have been registered to the UK Clozapine Central Non-Rechallenge Database, suggesting that Black individuals with undiagnosed BEN are being inappropriately included in the register, and with adverse consequences for their access to clozapine treatment.

Among other clinical risk factors, WCC spikes, although initially considered a potential early warning sign, have demonstrated poor specificity and predictive value in subsequent analyses (Alvir et al., 1995). While spikes during titration are assumed to reflect early inflammatory changes triggered by clozapine and/or its metabolites (Sernoskie et al., 2022), our results do not support the notion that this represents a priming process that increases the risk of later agranulocytosis. Similarly, febrile episodes and inflammatory syndromes may signal underlying immune dysregulation associated with clozapine use and have been linked with development of eosinophilia and neutropenia (Maes et al., 1997; Shao et al., 2024) but are not reliable early warning signs.

Collectively, these findings support the hypothesis that true clozapine-induced agranulocytosis is an idiosyncratic reaction, unrelated to demographic or baseline biological characteristics.

Clozapine dose and plasma concentration were not associated with increased agranulocytosis risk; if anything, higher concentrations appeared protective in some studies – a finding supported by our meta-analysis. One possible explanation is that neutropenic events tend to occur earlier in treatment, often during titration, although this was not reflected in our data, where two of the three included studies reported a mean time to event greater than 12 weeks (Matsui et al., 2020; Yagcioglu et al., 2016a). Another possible explanation could be clinicians’ reluctance to increase clozapine dose in patients with early hematological abnormalities, although this is not recommended by current guidelines. Importantly, these findings argue against adjusting clozapine dose thresholds as a preventative strategy.

Concomitant psychotropic medication is more consistently implicated. Valproate, particularly at higher doses, and antipsychotic polypharmacy were both associated with increased risk of agranulocytosis, based on qualitative synthesis of studies reporting on these risk factors. The mechanism behind valproate’s hematological toxicity remains unclear, including whether its effect on neutrophil counts is independent of clozapine or arise from an interaction between the two drugs, but the replication of this association highlights the importance of regularly reviewing co-prescribed medications in clozapine-treated individuals. It should be noted that no information on adherence was available in the included studies.

Interestingly, a recent meta-analysis (Myles et al., 2018) found no reduction in agranulocytosis rates following the introduction of mandatory monitoring in 1990, suggesting that early discontinuation in response to mild neutropenia may not prevent severe cases. Instead, emerging evidence suggests that true clozapine-induced agranulocytosis follows a characteristic pattern: a rapid ANC decline over ~2 weeks to below 0.5/mm^3^ for two consecutive days (Oloyede et al., 2024; Taylor et al., 2024). Such patterns may offer a more specific marker of clozapine-related events than isolated mild abnormalities.

### Other blood dyscrasia

For neutropenia and other blood dyscrasias, gender and age have again shown inconsistent associations. While some studies suggested higher incidence among women, others did not, and our meta-analyses found no significant gender effect, though the direction of association was toward increased risk in females. Similarly, age has not been reliably linked to neutropenia risk, with some evidence pointing to increased vulnerability in older adults, possibly reflecting age-related factors such as comorbidities, reduced bone marrow reserve, or increased peripheral white cell destruction – factors not necessarily linked to clozapine use (Ibáñez, Vidal, Ballarín, & Laporte, 2005). Notably, a within-subject design study (Johannsen et al., 2022) found no increased risk with advancing age.

Ethnicity-related findings are again difficult to interpret in the absence of systematic BEN assessment. Afro-Caribbean and African individuals more frequently presented with pre-treatment neutropenia and minor neutropenic events, almost certainly due to higher BEN prevalence, rather than clozapine-specific risk.

Among concomitant medications, valproate use has been associated with neutropenia and eosinophilia, suggesting a possible immune-mediated mechanism, although findings remain inconsistent. Conversely, lithium was associated with leukocytosis in a dose-dependent manner, consistent with its well documented effect on neutrophil count.

Baseline hematological parameters – particularly low WCC and neutrophil count – emerged as predictors of neutropenia, echoing the findings for agranulocytosis. A recent study (Goldani et al., 2022) examining neutropenia risk among both clozapine users and non-users found that higher baseline ANC was protective against subsequent neutropenic events, regardless of clozapine exposure. These results suggest that such associations may reflect underlying individual variability rather than clozapine-specific effects.

Febrile episodes and inflammatory syndromes were again observed in association with eosinophilia and neutropenia and may signal underlying immune dysregulation associated with clozapine use (Maes et al., 1997; Shao et al., 2024). However, as with agranulocytosis, they lack specificity and cannot be considered reliable predictors of blood dyscrasia.

### Clinical implications

Taken together, these findings support a shift from rigid universal monitoring protocols toward a more risk-stratified approach. Patients with persistently low baseline ANC, medical comorbidities, or concomitant medications that impair hematopoiesis may benefit from closer monitoring during the early phases of clozapine treatment. In contrast, long-term clozapine users without prior hematological abnormalities may be suitable for less intensive monitoring. Current data suggest that decisions about relaxed long-term monitoring should be guided primarily by the temporal decline in risk rather than by most patient-level predictors, with the potential exception of those with very low baseline ANC. This is consistent with evidence that agranulocytosis risk falls sharply after the first 12 months and with broader data showing that certain medical conditions (e.g. malignancy, HIV, viral hepatitis) increase neutropenia risk independently of clozapine use (Goldani et al., 2022). Additionally, given the limited consideration of BEN in the included studies, future research should systematically account for BEN, through ethnicity stratification, baseline neutrophil measurements, or genetic characterization, to improve the accuracy and clinical applicability of risk prediction models. Clinicians should likewise consider BEN when assessing neutropenia risk, including the potential role of ACKR1 genotyping prior to clozapine initiation, as identification of the homozygous variant associated with BEN may help avoid unnecessary treatment interruption (Aziri, Vallianatou, Balgobin, & Taylor, 2025; Murtough et al., 2026).

## Limitations

This study has several limitations. Firstly, a comprehensive analysis of certain risk factors for clozapine-associated blood dyscrasias was constrained by inconsistent reporting across primary studies. Secondly, heterogeneity in the definitions of blood dyscrasia types precluded their inclusion in the meta-analyses. Additionally, most of the included studies did not investigate or rule out alternative causes of blood dyscrasia, especially neutropenia, which may have confounded the findings and reduced their specificity. In fact, the variability in neutropenia definitions across studies prevented a systematic distinction between agranulocytosis and milder forms of neutropenia, which may have contributed to obscuring potential associations. Substantial heterogeneity was observed across several meta-analyses, likely reflecting differences in study design, population characteristics, definitions of blood dyscrasia, study periods, cohort selection, geographic settings, and other unmeasured methodological factors. This heterogeneity limits the reliability of pooled estimates for some risk factors, particularly age, where *I* ^2^ exceeded 85% across neutropenia analyses. Additionally, the methodological approaches of the included studies carry inherent risks of bias, and most did not systematically adjust for potential confounders. As a result, residual confounding may have contributed to overestimation or underestimation of certain associations. We also did not systematically search clinical trial registries nor accessed the manufacturer’s pharmacovigilance database and the Periodic Safety Update Reports, which may have limited the identification of unpublished data. Finally, given the widely reported association between clozapine and neutropenia and the presence of varying rigorous monitoring protocols across countries, the potential for observation bias is substantial in several included studies.

## Conclusion

This review suggests that although some factors may modestly influence the risk of clozapine-associated blood dyscrasia, no single strong predictor has been identified. The substantial heterogeneity across studies, particularly for some risk factors, limits the confidence with which these findings can be interpreted. Our meta-analyses showed a positive association between neutropenia and concomitant psychotropic use, and a modest inverse association between agranulocytosis and both clozapine dose and baseline WCC. However, these latter findings do not necessarily indicate real protective effects against true clozapine-induced agranulocytosis. Additionally, failure to systematically account for BEN may have led to misleading associations, highlighting the need for its careful consideration in future studies. The considerable variability across studies underscores the limitations of current evidence, much of which is derived from retrospective analyses or pharmacovigilance data vulnerable to under-reporting and confounding.

## Supporting information

10.1017/S0033291726104371.sm001 Casetta et al. supplementary material Casetta et al. supplementary material

## Data Availability

The data that support the findings of this study are available on request from the corresponding author, CC, upon reasonable request.

## Supplementary material

The supplementary material for this article can be found at http://doi.org/10.1017/S0033291726104371.
