# Supplementary material for: Risk factors associated with blood dyscrasia during clozapine treatment: a systematic review and meta-analysis
Source: Psychol Med. 2026 May 11;56:e142. doi: 10.1017/S0033291726104371 (PMC13161814; doi:10.1017/S0033291726104371)
Supplement: Casetta et al. supplementary material [file S0033291726104371sup001.docx]

| **NEWCASTLE - OTTAWA QUALITY ASSESSMENT SCALE CASE** | | | | | | | | | | | | | |
| --- | --- | --- | --- | --- | --- | --- | --- | --- | --- | --- | --- | --- | --- |
| **Author** | **Year** | Selection 1 | Selection 2 | Selection 3 | Selection 4 | Comparability | Outcome 1 | Outcome 2 | Outcome 3 | Selection TOTAL | Comparability TOTAL | Outcome TOTAL | Overall quality (AHRQ standards) |
| Hummer | 1992 | 1 | 1 | 1 | 1 | 0 | 1 | 0 | 1 | 4 | 0 | 2 | Poor |
| Lieberman | 1992 | 1 | 1 | 1 | 1 | 1 | 1 | 1 | 1 | 4 | 1 | 3 | Good |
| Alvir | 1993 | 1 | 1 | 1 | 1 | 1 | 1 | 0 | 1 | 4 | 1 | 2 | Good |
| Banov | 1993 | 1 | 1 | 1 | 1 | 0 | 1 | 1 | 1 | 4 | 0 | 3 | Poor |
| Alvir | 1994 | 1 | 1 | 1 | 1 | 1 | 1 | 0 | 1 | 4 | 1 | 2 | Good |
| Hummer | 1994 | 1 | 1 | 1 | 1 | 0 | 1 | 0 | 0 | 4 | 0 | 1 | Poor |
| Alvir | 1995 | 1 | 1 | 1 | 1 | 1 | 1 | 0 | 1 | 4 | 1 | 2 | Good |
| Atkin | 1996 | 1 | 1 | 1 | 1 | 0 | 1 | 0 | 1 | 4 | 0 | 2 | Poor |
| Mauri | 1998 | 0 | 1 | 1 | 1 | 0 | 1 | 0 | 1 | 3 | 0 | 2 | Poor |
| Copolov | 1998 | 1 | 1 | 1 | 1 | 0 | 1 | 1 | 0 | 4 | 0 | 2 | Poor |
| Munro | 1999 | 1 | 1 | 1 | 1 | 0 | 1 | 1 | 1 | 4 | 0 | 3 | Poor |
| Lambertenghi | 2000 | 1 | 1 | 1 | 1 | 0 | 1 | 1 | 0 | 4 | 0 | 2 | Poor |
| Dettling | 2001 | 1 | 1 | 1 | 1 | 1 | 1 | 1 | 0 | 4 | 1 | 2 | Good |
| Kang | 2006 | 1 | 1 | 1 | 1 | 0 | 1 | 1 | 0 | 4 | 0 | 2 | Poor |
| Dunk | 2006 | 1 | 1 | 1 | 1 | 0 | 1 | 1 | 0 | 4 | 0 | 2 | Poor |
| Maher | 2013 | 1 | 1 | 1 | 1 | 0 | 1 | 0 | 1 | 4 | 0 | 2 | Poor |
| Abanmy | 2014 | 1 | 1 | 1 | 1 | 0 | 1 | 0 | 0 | 4 | 0 | 1 | Poor |
| Balda | 2015 | 1 | 1 | 1 | 1 | 1 | 1 | 1 | 0 | 4 | 1 | 2 | Good |
| Lau | 2015 | 1 | 1 | 1 | 0 | 0 | 1 | 1 | 0 | 3 | 0 | 2 | Poor |
| Meyer | 2015 | 1 | 1 | 1 | 1 | 0 | 1 | 1 | 1 | 4 | 0 | 3 | Poor |
| Demler | 2016 | 1 | 1 | 1 | 0 | 0 | 1 | 0 | 0 | 3 | 0 | 1 | Poor |
| Yağcıoğlu | 2016 | 1 | 1 | 1 | 1 | 0 | 1 | 1 | 1 | 4 | 0 | 3 | Poor |
| Prokopez | 2016 | 1 | 1 | 1 | 1 | 0 | 1 | 1 | 0 | 0 | 0 | 2 | Poor |
| Fabrazzo | 2017 | 1 | 1 | 1 | 1 | 0 | 1 | 1 | 0 | 4 | 0 | 2 | Poor |
| Vargas | 2017 | 1 | 1 | 1 | 1 | 0 | 1 | 1 | 1 | 4 | 0 | 3 | Poor |
| Hollingworth | 2017 | 1 | 0 | 0 | 0 | 0 | 0 | 1 | 1 | 1 | 0 | 2 | Poor |
| Malik | 2018 | 1 | 1 | 0 | 1 | 1 | 1 | 1 | 1 | 3 | 1 | 3 | Good |
| Royer | 2019 | 1 | 1 | 1 | 1 | 0 | 1 | 1 | 0 | 4 | 0 | 2 | Poor |
| Tunsirimas | 2019 | 1 | 1 | 1 | 0 | 0 | 1 | 1 | 0 | 3 | 0 | 2 | Poor |
| Mena | 2019 | 1 | 1 | 1 | 0 | 1 | 1 | 1 | 1 | 3 | 1 | 3 | Good |
| Matsui | 2020 | 1 | 1 | 1 | 1 | 1 | 1 | 1 | 1 | 4 | 1 | 3 | Good |
| Toyoda | 2021 | 1 | 1 | 1 | 0 | 0 | 1 | 0 | 0 | 3 | 0 | 1 | Poor |
| Imazu | 2021 | 1 | 1 | 1 | 0 | 0 | 1 | 0 | 0 | 3 | 0 | 1 | Poor |
| Tsukiji | 2021 | 1 | 1 | 1 | 1 | 1 | 1 | 0 | 1 | 4 | 1 | 2 | Good |
| Gee | 2021 | 1 | 1 | 1 | 1 | 0 | 1 | 1 | 1 | 4 | 0 | 3 | Poor |
| Johannsen | 2022 | 1 | 1 | 1 | 1 | 1 | 1 | 1 | 1 | 4 | 1 | 3 | Good |
| Glocker | 2023 | 1 | 1 | 1 | 0 | 0 | 1 | 0 | 0 | 3 | 0 | 1 | Poor |
| Yang | 2023 | 1 | 1 | 1 | 0 | 1 | 1 | 1 | 0 | 3 | 1 | 2 | Good |
| Uwai | 2023 | 1 | 1 | 1 | 0 | 1 | 1 | 0 | 0 | 3 | 1 | 1 | Poor |
| Kang | 2023 | 1 | 1 | 0 | 0 | 1 | 1 | 0 | 0 | 2 | 1 | 1 | Poor |
| Northwood | 2024 | 1 | 1 | 1 | 1 | 0 | 1 | 1 | 1 | 4 | 0 | 3 | Poor |
| Rubio | 2024 | 1 | 1 | 1 | 1 | 1 | 1 | 1 | 1 | 4 | 1 | 3 | Good |
| Bleich | 2024 | 1 | 1 | 1 | 0 | 0 | 1 | 0 | 0 | 3 | 0 | 1 | Poor |
| Kikuchi | 2024 | 1 | 1 | 1 | 1 | 1 | 1 | 1 | 1 | 4 | 1 | 3 | Good |

*Supplementary table 1. Quality assessment of included studies (Newcastle - Ottawa Quality Assessment Scale Case)*
